# Zoonotic Parasites in Artiodactyls with Emphasis on the Feral Boar in the Atlantic Forest, State of Rio de Janeiro, Brazil

**DOI:** 10.3390/ani13233611

**Published:** 2023-11-22

**Authors:** Jessica L. Pinheiro, Sávio F. Bruno, Laís V. Dib, Claudijane R. Dos Santos, Camila S. C. Class, Laís L. Corrêa, Marcelo Studart Lima, Paulo Rogério A. Motoyama, Ricardo J. P. S. Guimarães, Maria Regina R. Amendoeira, Alynne S. Barbosa

**Affiliations:** 1 Laboratório de Bioagentes Ambientais, Departamento de Microbiologia e Parasitologia, Instituto Biomédico, Universidade Federal Fluminense, Niterói 24210-130, RJ, Brazil; limajessica@id.uff.br (J.L.P.); camilaclass@id.uff.br (C.S.C.C.); laislisboa@id.uff.br (L.L.C.); 2 Setor de Medicina de Animais Selvagens, Faculdade de Veterinária, Universidade Federal Fluminense, Niterói 24230-340, RJ, Brazil; saviobruno@id.uff.br; 3 Laboratório de Protozoologia, Instituto Oswaldo Cruz, Fundação Oswaldo Cruz, Rio de Janeiro 21040-360, RJ, Brazil; laisvdib@gmail.com (L.V.D.); claudijaneramos@id.uff.br (C.R.D.S.); 4 Faculdade de Medicina de Campos, Campos dos Goytacazes, Rio de Janeiro 28035-581, RJ, Brazil; 5 Parque Estadual da Pedra Selada, Visconde de Mauá 27553-970, RJ, Brazil; mstudart89@gmail.com (M.S.L.); prmotoyama.peps@gmail.com (P.R.A.M.); 6 Laboratório de Geoprocessamento, Instituto Evandro Chagas, Ananindeua 67030-000, PA, Brazil; ricardojpsg@gmail.com

**Keywords:** feral boar, zoonotic parasites, gastrointestinal parasites, *Balantioides coli*, conservation unit

## Abstract

**Simple Summary:**

The present study reported a series of parasites in the feces of free-living feral boar collected in a conservation unit with an Atlantic Forest biome in Rio de Janeiro, Brazil. Several of the parasites detected showed zoonotic transmission potential, that is, they can be transmitted to other animals, including humans. Although the samples were taken directly from the park environment (trails), the identification of the host species was confirmed from the molecular diagnosis. Some of the feces of the feral boar bioinvader were collected in the specific area of the park that is closest to human homes. It is worth noting that *Balantioides coli* parasite was characterized in the feces of these animals, which can be transmitted to humans from contaminated water, fruits, and vegetables and cause dysentery in people, which can lead to death.

**Abstract:**

Background: The purpose of this study was to identify the species of artiodactyl host related to the fecal matter collected in a forest area in Rio de Janeiro state and carry out a parasitological investigation. Methods: Artiodactyl feces were collected between 2020 and 2021. The fecal samples were examined to identify the host through macroscopic and molecular analysis. The remaining samples were subjected to a fecal parasite analysis using microscopic techniques, and feces containing cysts of the phylum Ciliophora underwent a molecular analysis. Results: Of the 101 samples collected, 71.3% were found in Pavão Valley, the most anthropized area of the park. In the molecular analysis, 79 samples were identified as belonging to *Sus scrofa* and 2 as *Mazama gouazoubira*. The most frequently detected forms were cysts of the phylum Ciliophora (39.6%), followed by eggs of *Ascaris* spp. (11.8%) and *Metastrongylus* spp. (5.9%). Nucleotide sequences of *Balantioides coli* were characterized in 26 samples, and in 13 samples variants of type B0 and in 11 type A0 were identified. Conclusions: It should be noted that this is the first study in the Americas that has identified *B. coli* in free-living *S. scrofa* feces, citing this bioinvader as one of the reservoirs of this parasite.

## 1. Introduction

The native wild artiodactyls that circulate freely in the Atlantic Forest biome in Brazil are *Tayassu tajacu*, *Tayassu pecari*, and *Mazama gouazoubira*. These animals play a crucial role in seed dispersal in the environment [1]. In addition to the artiodactyls native to Brazil’s fauna, the family Suidae, represented by the wild boar *Sus scrofa* and the domestic pig, *Sus scrofa domesticus*, has also been reported in the wild [2]. *Sus scrofa* is considered one of the most impactful exotic bioinvasive species due to its aggressiveness, adaptability, uncontrolled reproduction, and absence of predators [3,4]. Among the exotic mammals that occur in Brazil today, these animals are the species most widely distributed in the country [5]. In Brazil, *Sus scrofa* was introduced in the 1960s for the main purpose of meat production. Albeit popularly known as feral boar, the individuals that live in its wild form are generally a crossbreed of the European wild boar with domestic pigs, thus giving rise to the popular Brazilian designation of “javaporco” (a contraction of the words javali [wild boar] and porco [pig]) [3,4].

Over the years, the wild mammal fauna has been decreasing more and more around the world due to several factors, including parasitism by different etiological agents that can be introduced into the wild environment by exotic, bioinvasive animals due to anthropogenic changes [6,7]. When parasitized the animals can present weight loss, metabolic imbalance, reproductive problems, anemia, and dehydration, and, in more severe cases, the death of the animal can occur [8]. Furthermore, parasitic infections can determine behavioral and functional changes in wild animals in their niche [9].

Many of these parasites that infect pigs and other artiodactyls can be transmitted to humans, determining zoonotic transmission cycles, as *Ascaris* spp., *Metastrongylus* spp., *Entamoeba suis*, and *Balantioides coli* [10,11,12,13,14]. In this context, the protozoan *B. coli* stands out, which has domestic and wild pigs as its main reservoir [15]. The transmission of this parasite occurs mainly from environmental contamination with the cystic form through the ingestion of contaminated water, fruits, and vegetables [12,16]. Clinical manifestations of human balantidiasis can range from diarrhea to more serious invasive clinical conditions such as dysentery, which can lead to death. Furthermore, there is still no fully effective treatment for eliminating *B. coli* infection [12,17]. Therefore, to minimize the transmission of these zoonotic pathogens in ecological wild areas, it is extremely important that researchers act within a One Health approach, that is, with the monitoring of pathogens recovered from environmental samples where animals and humans circulate to provide solid information for prophylactic measures and the control of possible illnesses [18].

Wild artiodactyl species native to Brazil are known to circulate in this country, but there is still little information about bioinvasive artiodactyls and the parasites that infect them. Therefore, it is essential to monitor the fauna and parasitic agents of these animals constantly, especially in protected areas, to prevent possible ecological and health imbalances, particularly in highly degraded biomes such as the Atlantic Forest in which parasitological monitoring was never carried out. In view of the above, this study focused on identifying the species of artiodactyl hosts related to fecal matter collected non-invasively in a forest conservation unit of the Atlantic Forest biome in the state of Rio de Janeiro, carrying out a parasitological investigation of the feces of these animals and a molecular characterization of the protozoa of the phylum Ciliophora detected to identify the zoonotic protozoan *B. coli.* The resulting information was associated with the areas of the forest in which these animals roam to assess the presence of the animal species and the possible impacts of the parasitic agents identified in its fecal matter.

## 2. Materials and Methods

### 2.1. Fecal Sample Collection Site

**Figure 1:**
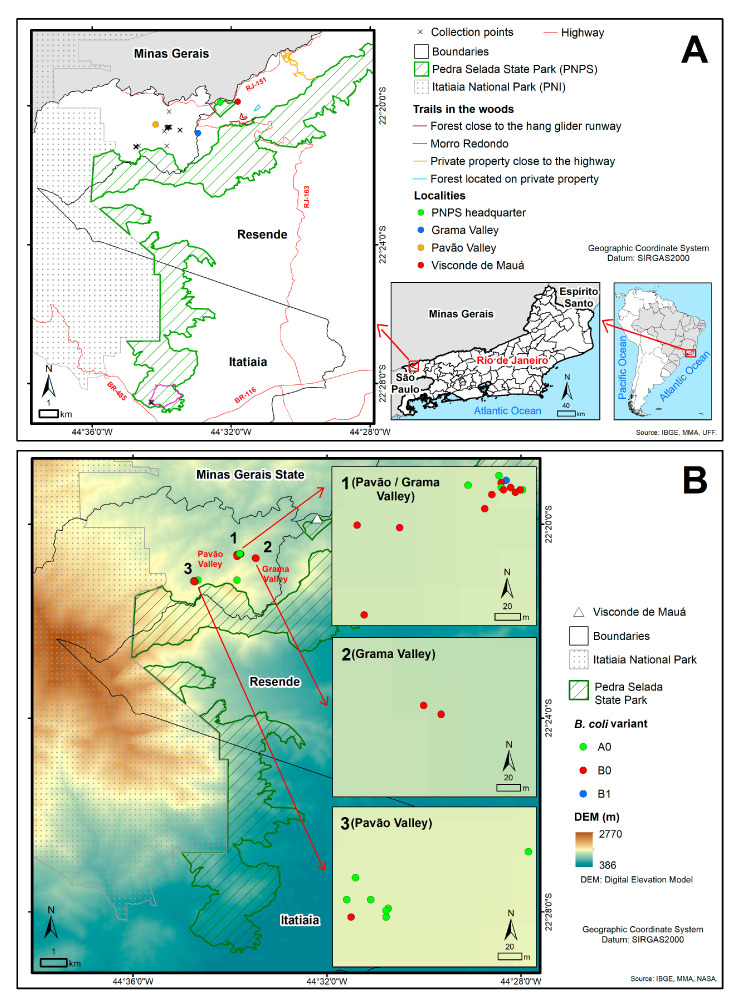
(**A**) Identification of the study area, pinpointing the location of samples collected in the field. (**B**) Spatial distribution of the genetic variant of *Balantioides coli* detected in fecal samples collected in Grama and Pavão Valleys. Images A and B show the spatial location of variants of *B. coli* on a larger scale. Source: The author.

Fecal samples were collected in Pedra Selada State Park, which is located in Itatiaia and Resende cities Rio de Janeiro state. The Park covers an area of approximately 8036 hectares, cutting across the Resende and Itatiaia cities, located in the middle Paraíba Valley region and the Mantiqueira mountain in the south of Rio de Janeiro. This conservation unit protects an important area of the Atlantic Forest biome, at altitudes ranging from 600 to 2100 m above sea level (MASL). The main tourist attraction in the park is Pico da Pedra Selada, which is 1755 MASL [19]. The park is the only state conservation unit in Mantiqueira mountain, enabling the formation of vital ecological corridors that protect the headsprings in some of the main river basins in Brazil’s Southeast Region. The region protected by the park comprises a wide forest corridor containing rural summer houses. The region has a high elevation subtropical climate, with mild average temperatures. The lowest mean temperature is 11 °C, but it may go down to −9 °C in June. The park is located in a region that is home to several endemic and endangered species, including birds, amphibians, and mammals. [19] The level of difficulty of the areas covered here is very high, since they actually consist of steep terrain with dense forest, without delimited trails. The areas covered are locally known as Pavão Valley, Grama Valley, Forest located on private property close to the highway, Forest close to the hang glider runway, electrical towers, and Morro Redondo (Round Hill) (Figure 1A).

### 2.2. Collection of Fecal Samples

Fecal samples were collected between July 2020 and August 2021, prioritizing a collection period of 12 months, in locations of the park where the wildlife was previously being monitored by park rangers using camera traps, as well as through visual records reported by residents. 

All these routes were covered on foot, totaling around 80 km covered, and were always carried out with the presence of at least one park ranger responsible for monitoring the local fauna. The sections of the places mentioned above were explored only once, and they were carried out very slowly looking for feces, that is, exploring the environment, as artiodactyls feces are generally found in areas of dense forest. The sections were always covered in the coldest and driest months of the year to avoid the leaching of fecal samples and to avoid exposing the team to dangerous situations due to rain. In total, three fieldworks were carried out in Pavão Valley, three in Grama Valley, two in Morro Redondo, two in a forest located on private property close to the highway, and two in a forest close to a hang-gliding track and electrical towers.

**Figure 2:**
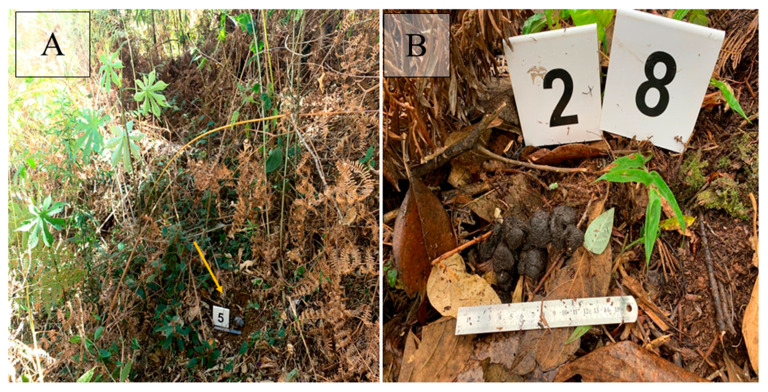
Identification of samples during field sampling: (**A**) sample collected on the first day of collection in Pavão Valley; yellow arrow pointing to fecal sample. (**B**) Fecal sample number 28 collected on the third day in Pavão Valley. Source: the author.

The sites where each fecal sample was found were georeferenced, using a Garmin Etrex 20× GPS. At the collection points, the fecal samples were identified with plastic number plates and photographed with a ruler to provide a sense of scale. As a criterion, priority was given to recovering feces that had an intact shape, that is, that were not trampled or crushed. They were then stored in plastic bags without a chemical preservative, containing the respective identification number (Figure 2A,B). In addition, the identification number, date, time, and place of collection and the georeferencing points for each sample were recorded on technical data sheets. The collected samples were sent to the laboratory in cooler boxes. In the laboratory, the samples were kept refrigerated at 4 °C until analysis.

### 2.3. Host Identification—Macroscopic Morphological Analysis

The first step taken to identify the host species that produced the fecal matter was to make a macroscopic analysis of feces based on observation and a comparison of data published in the literature. This analysis followed a standard procedure [20]. As soon as it arrived at the laboratory, not exceeding 24 h after collection, the fecal samples were first weighed on an analytical scale, after the material was deposited on a white sheet to record its color, presence of artifacts and possible components of the diet, and to measure the length and diameter of all the fecal propagules was carry out with pachymeter. Each sample was then photographed with an identification tag at the top and a ruler at the bottom to provide a sense of scale. All the resulting information was compared with taxonomic classification guides [21,22].

### 2.4. Host Identification—Molecular Analysis

The samples were also subjected to a molecular analysis to identify the host species related to the fecal matter. After filtering the fecal samples through a sieve and four folded gauzes into a conical bottom analyzer flask, half of the sediment was aliquoted in microtubes. The DNA of these samples was extracted using a PureLink Microbiome DNA extraction kit (Invitrogen^®^, Waltham, MA, USA), as recommended by the manufacturer.

After extraction, the DNA was subjected to polymerase chain reaction (PCR). This procedure involved adding the following products to each 0.2 mL microtube: 1.2 µL of Tris-KCl 1× buffer (Tris-HCl 20 mM, pH 8.4, and KCl 50 mM), 0.5 µL of MgCl_2_ (50 mM) (Invitrogen™), 0.8 µL of dNTPs (2.5 nM of each dNTP), 1 µL of BC-F2 forward primer (5′ ATCACCACTATTGTTAATATAAAACC 3′), and 1 µL of HCO-2198 reverse primer (5′ TAAACTTCAGGGTGACCAAAAAATCA 3′) at 1 pmol each, which amplified the COI region with about 239 base pairs of mitochondrial DNA from animals of different orders described in the literature [23]. We also used 0.2 µL of Taq polymerase (1 IU, Invitrogen™ Platinum^®^ Taq DNA Polymerase), 0.3 µL of BSA (0.2 nM—Bovine serum albumin), and 5 µL of the extracted DNA. PCR was performed in an Applied Biosystems thermal cycler (Life Technologies^®^, Carlsbad, CA, USA) following the standard procedure described in the literature. The amplified product was examined in 1.5% agarose gel electrophoresis with the addition of GelRed (Biotium^®^, Fremont, CA, USA) and bromophenol blue solution (LGC^®^, Teddington, UK). Negative controls consisting of ultrapure water, and positive controls corresponding to a fecal sample of an artiodactyl from other laboratory projects, were used in all the extraction and polymerase chain reaction steps. 

The PCR products were purified using ExoSAP-IT enzyme (Invitrogen^®^), and all the samples were sequenced in an Applied Biosystems 3730 DNA Analyzer on the platform of CREBIO, the Center for Biological Resources and Genomic Biology at São Paulo State University (UNESP) in Jaboticabal, and in the Microbiology and Parasitology Multiuser Laboratory of the Biomedical Institute at Fluminense Federal University (UFF). The resulting nucleotide sequences were aligned and edited using BioEdit version 7.2.5 software and then compared with the MN608176.1 reference sequences [20,24,25] of artiodactyl species deposited in the GenBank database. 

### 2.5. Microscopic Parasitological Diagnosis

Half of the fecal material was removed to perform microscopic parasitological techniques. Part of the fecal filtrate deposited in the conical bottom flask was aliquoted in 15 mL conical centrifuge tubes for the qualitative parasitological techniques of feces examination by centrifugal sedimentation with ethyl acetate, which has two centrifugation steps, at 2000 rpm for 2 min [26,27], centrifugal flotation with sucrose, which has two centrifugation steps, one with fecal filtrate at 1500 rpm for 10 min, and the other with sucrose solution at 1500 rpm for 5 min [28,29] and spontaneous sedimentation [30]. 

The microscopy slides obtained from each technique and the photomicrograph were read in an Olympus BX41 microscope, firstly under approximately 100× magnification, and for confirmation, when necessary, 400×. The microscope was coupled to a Samsung SDC415 digital camera with Honestech TVR recording software, version 2.0.

### 2.6. Molecular Analysis of Ciliated Protozoa

All the fecal samples positive for protozoan cysts compatible with the phylum Ciliophora were subjected to molecular analysis for the taxonomic identification of the parasite, as well as its genetic variant. 

The fecal material was placed on a stereoscopic microscope, and the cysts were manually removed using a glass Pasteur pipette and transferred to 1.5 mL microtubes, totaling an average of 30 cysts per sample. The material was then centrifuged at 14,000 rpm for 1 min, and the material was maintained at a final volume of approximately 50 µL as previously standardized [31]. The microtubes were then placed at refrigerated (4 °C) temperature, and the next day, they were subjected to DNA extraction with the commercial kit QIAamp^®^ Fast DNA stool mini kit (QIAGEN) following the manufacturer’s recommendations. Two modifications were implemented. First, at the beginning of the extraction step, the material was subjected to three cycles of thermal shock. This involved placing the microtubes in liquid nitrogen (−196 °C) for three minutes and then in a dry bath incubator at 95 °C for three minutes. In addition to this modification, the incubation step (70 °C) in the dry bath incubator with lysis buffer and proteinase was increased to 24 h. After extraction, the DNA was stored in a freezer.

The DNA extracted from the samples was subjected to PCR using a Platinum Hot-Start PCR Master Mix (Invitrogen^®^) and B58D and B58RC primers that amplify the DNA fragment from the ITS1.5.8S.ITS2 region of ciliated protozoa, as described in the literature [32]. The amplified product was purified with ExoSAP-IT enzyme (Invitrogen^®^) and sequenced on the CREBIO platform. 

The analysis and initial editing of the sequences was performed using ChromasPro version 1.7.5 software. The sequences were saved in FASTA format and aligned with other homologous sequences taken from the GenBank database using BioEdit software. Phylogenetic inferences were obtained from maximum likelihood, and the best evolutionary model was selected based on the Akaike information criterion (AIC) using the IQ-TREE web server. Editing of the phylogenetic tree, as well as rooting, were performed using MEGA-X software, version 11. Sequences were selected from the Genbank of *Balantioides coli* and other species of ciliated protozoa to compose the analyses [33,34,35,36]. 

### 2.7. Analysis of the Results

The georeferenced fecal sample collection points were plotted on park maps produced with ArcGis version 10.4 software, together with data from a digital elevation model (DEM) obtained from SRTM [37]. 

The frequency of parasites was determined by dividing the number of positive samples by the total number of collected samples, taking into account identified and unidentified host species. Fisher’s exact test was used to determine the statistical relevance of the parasite taxon in relation to the number of collected samples. This analysis was performed using Epi Info™ software version 7.2.5.0, CDC, (Atlanta, GA, USA) with a 5% significance level. In addition, the biological agents were counted according to the parasitic forms, and this count was included in the indices of richness, diversity, and dominance. These statistical tests were analyzed using Microsoft Excel^®^ version 365 and Past^®^ version 3.2.2 software [38]. 

To verify the parasite richness in the taxon of the identified artiodactyl in relation to its species and to analyze sampling sufficiency, parasite accumulation graphs were created [39]. These graphs were also produced considering the main sample collection sites. Parasite diversity, richness, and dominance were compared among the main collection sites according to the species of artiodactyl identified. The Shannon and Simpson indices were used for a statistical analysis of parasite diversity [40]. 

Lastly, the molecular characterization of the protozoan of the phylum Ciliophora was associated with the identification of the host. In addition, the collection points of the samples positive for the protozoan species of the phylum Ciliophora and the characterized variants were plotted on the park map, according to the areas covered, to determine the distribution of these parasites in the environment.

## 3. Results

Of the 101 fecal samples collected, 72 (71.3%) were found in the areas traversed in Pavão Valley, 24 (23.8%) in Grama Valley, and 5 (4.9%) in Morro Redondo. No fecal samples were found in the areas of private property or in the proximity of the hang glider runway (Figure 1). Of the 101 fecal samples collected, all underwent PCR and DNA sequencing to identify the host related to the fecal matter through molecular analysis using primers that amplify a DNA fragment of the COI gene. Fragments of nucleotide sequences were identified in 81 samples, which aided in the interpretation of the host species, the vast majority of which, i.e., 79, were compatible with *S. scrofa* (feral boar) with an identity value greater than 99% vis à vis the reference sequence. Only two nucleotide sequences from fecal samples collected in Morro Redondo showed 86.7% identity with the *Mazama gouazoubira* sequence deposited in GenBank, but they presented no degree of identity compatible with any other artiodactyl species when searched with the BLASTn tool. All reference sequences were deposited in the Dryad Data Repository at https://doi.org/10.5061/dryad.q573n5tmd (accessed on 2 June 2022).

Parasite forms were detected in 54 (53.4%) of the 101 fecal samples collected in the areas of Pedra Selada State Park, RJ, as well as in its buffer zones (BZs) (Table 1). Protozoa, which were detected in 41 (40.5%) fecal samples, were found more frequently than helminths, which were identified in 28 (27.7%) samples. Cysts and oocysts compatible with the phyla Ciliophora and Apicomplexa, respectively, were identified among the protozoa, while only forms of the phylum Nematoda were found among the helminths.

The most common forms identified were rounded brownish to yellowish cysts with an average diameter ranging from 101.45 µm (±26.6) to 105.3 µm (±48.9) compatible with the cysts of protozoa of the phylum Ciliophora, which were found in 40 (39.6%) fecal samples. This positivity rate was statistically significant within the total number of samples collected (*p* ≤ 0.05) (Table 1 and Table S1). 

These structures were most frequently visible in feces taxonomically identified as belonging to *S. scrofa* based on the analysis of the DNA fragment of the COI gene, as well as in those in which the host species was not taxonomically identified, designated in this study as an “UIS artiodactyl” (unidentified species). In general, cysts compatible with the phylum Ciliophora were the most frequently detected forms of parasites in both Pavão and Grama Valley (Supplementary S1). In addition to this protozoan, unsporulated coccidian oocysts were also detected in the feces of *S. scrofa* 3 (5%) in Pavão Valley. However, the frequency of diagnosis of this structure was not statistically significant (*p* > 0.05) (Table 1).

The second most frequently identified parasite taxon was *Ascaris* spp., which was detected in 12 (11.8%) of the fecal samples (Table 1, Figure S1B). Eggs of this nematode were identified in artiodactyl feces found in all the areas of the park and in its buffer zones, i.e., in the feces of *S. scrofa* in nine samples in Pavão (15%) and two in Grama Valley (10.5%), and in a fecal sample identified as belonging to *M. gouazoubira* (100%) collected in Morro Redondo. Nematode larvae were also detected in the feces of *S. scrofa* in eight (7.9%) fecal samples. Moreover, six (5.9%) fecal samples were found to contain slightly yellowish ellipsoid eggs with thick and rough shells containing a single larva, with the smallest mean diameter being 68.4 µm (±34.6) and the largest one 95.2 µm (±44.9), which is typical of *Metastrongylus* spp. Of these positive samples for *Metastrongylus* spp., five (8.3%) belonged to *S. scrofa* and were collected in Pavão Valley. The frequencies of these nematodes were statistically significant in relation to the number of collected samples (*p* ≤ 0.05) (Table 1 and Figure S1E). 

Transparent thin-shelled strongyle eggs, bi-operculate tray-shaped eggs with the brownish coloration typical of *Trichuris* spp., and thick-shelled brownish nematode eggs containing a single taxonomically unidentified cell were only detected in the fecal samples identified in *S. scrofa* feces collected in Pavão Valley (Table 1, Figure S1D,F). 

**Figure 3:**
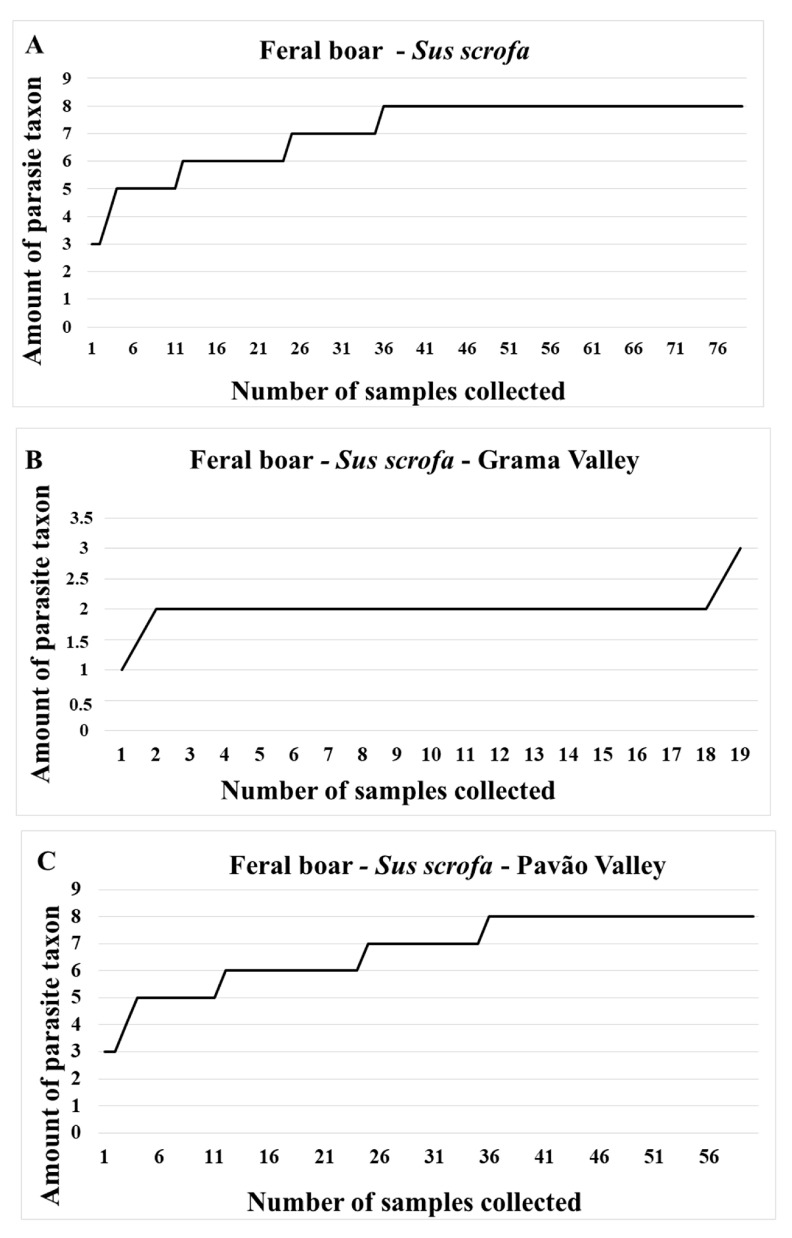
Accumulation curves of gastrointestinal parasites detected in fecal samples identified as *Sus scrofa* droppings collected in Pedra Selada State Park and its buffer zones, Rio de Janeiro. (**A**) general accumulation curve, taking into account all the collection points and samples identified as *Sus scrofa*. (**B**) Fecal samples collected only in Pavão Valley. (**C**) Fecal samples collected in Grama Valley.

In general, it was found that the parasite accumulation curve stabilized in about 36 fecal samples identified as *S. scrofa* (Figure 3 and Figure S1A). A curve stabilization plateau was also evidenced when the fecal samples of this host were analyzed considering only the samples collected in Pavão Valley (Figure 3 and Figure S1B). However, the curve pertaining to the feces identified as *S. scrofa* collected in Grama Valley (Figure 3 and Figure S1C) showed no evidence of stabilization. In the case of *M. gouazoubira*, a parasite accumulation curve could not be created because only two fecal samples were identified as belonging to this artiodactyl species.

With regard to the parasitic structures identified in the fecal samples of *S. scrofa* according to the collection sites, feces collected in Pavão Valley (R = 8) showed a greater richness of parasite forms than those from Grama Valley (R = 3). The latter exhibited greater parasite dominance, which was attributed to the phylum Ciliophora. The *S. scrofa* feces collected in Pavão Valley also showed higher parasite diversity by both the Shannon (H’) and Simpson indices (D), which showed higher statistical significance in the diversity *t* test, *p* ≤ 0.05, than those from Grama Valley (Table 2).

**Figure 4:**
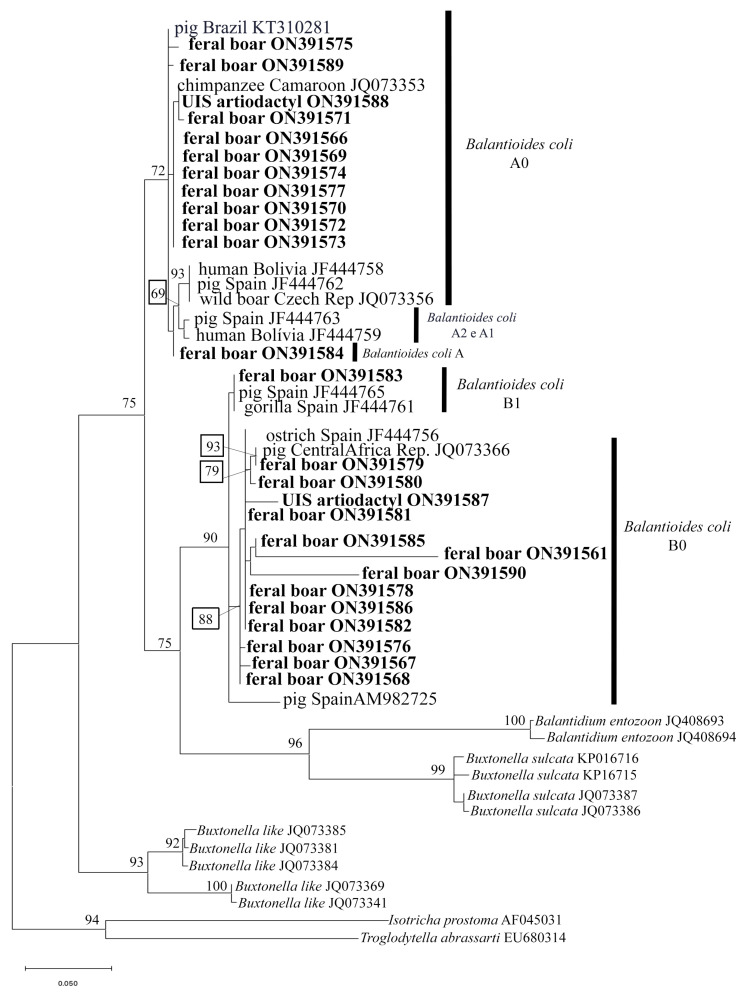
Phylogenetic tree based on the alignment of 370 bp (base pairs) of the DNA fragment of rRNA gene, fragment ITS1—5.8s rRNA—ITS2 of ciliated protozoa, using the maximum likelihood method with a GTR + G4 + F model of evolution. Sequences in this study are highlighted in bold, according to the genetic variant. Sequences from *Isotricha prostoma* and *Troglodytella abrassarti* are used as outgroups. The numbers associated with the branches refer to the bootstrap values for 1000 replications. UIS (unidentified species).

Of the 40 samples in which cysts similar to those of the phylum Ciliophora were detected by parasitological analysis, 32 showed bands of 500 bp in the amplification of DNA of the ITS1.5.8S.ITS2 fragment of rRNA. Of these, the generated nucleotide sequences were interpreted in 26 samples, and they were all deposited in GenBank under accession numbers ON391566 to ON391591. When compared with reference sequences deposited in GenBank, all these sequences were found to be compatible with *Balantioides coli*, with identity values ranging from 97% to 100%. The topography of the phylogenetic tree and an analysis of the nucleotide sequences indicated that 13 were classified as a genetic variant of type B0, 1 as type B1, and 11 as type A0 (Figure 4). Only one sequence at ON391584 was characterized as type A, although it did not fully match any pre-established subtype. This sequence exhibited 98.3% identity with types A1 and A2 and 98.9% with type A0.

In Pavão Valley, variant sequences were identified for *B. coli*, mostly of type A0 (11/26), followed by type B0 (5/26). In Grama Valley, sequences were identified, mainly of types B0 (8/26), type B1 (1/26), and one characterized only as type A (1/26) (Figure 1B).

With respect to the host, among the 26 samples in which *B. coli* was molecularly characterized, 24 were identified as belonging to *S. scrofa*, showing mainly the B0 type variant (12/24), followed by A0 (10/24), while only 2 were classified differently, namely, 1 as type B1 and the other as A (Figure 4).

## 4. Discussion

The priority setting of this research was to identify parasites in fecal samples collected directly in the environment, following a noninvasive convenience sampling method. This procedure resulted in the collection of 101 fecal samples of artiodactyls. Among the trails traversed in Pedra Selada State Park, a larger number of feces were collected in Pavão Valley. The larger number of fecal samples found in this valley appears to be due to the greater number of residences scattered around its perimeter, favoring sightings of these animals by residents, and hence the monitoring of this fauna by park rangers.

The noninvasive sampling method adopted in this study offers the advantage of preventing wild animals from having to endure the stressful and unnecessary experience of being captured and avoiding the need for researchers to find an animal carcass at random, which may be a time-consuming task [41]. However, the disadvantages of this procedure to obtain biological material are the researcher’s lack of knowledge about the host species and the number of individuals to which they belong. Nevertheless, this method of collecting biological material can be a suitable tool for studies involving elusive animals [42], including skittish and solitary animals that end up being predated and hunted more easily, as is the case of *M. gouazoubira* (gray brocket), a species of interest in this study.

In this study, the identification of the host began with a morphological analysis of the collected fecal samples in order to minimize the disadvantage of not knowing the host, i.e., the mammal associated with the fecal matter found in the forest. The fecal matter consisted of rounded pellets with one end slightly pointed, similar to descriptions in the literature for the order Artiodactyla [21,22].

Of the 101 samples collected, 79 were identified as belonging to *S. scrofa*. These samples were generally found in disturbed forest and trampled ground, containing several separate pieces of scat. Like in this forest park, *S. scrofa* has also been reported in parasitological surveys carried out in other conservation units in Brazil, Mexico, Spain, Portugal, and Iran [20,43,44,45,46,47,48]. Although other species of artiodactyls such as *Tayassu tajacu* (collared peccary) and *Tayassu pecari* (white-lipped peccary) have already been recorded on several occasions in the park, the predominance of *S. scrofa* as the host of most of the fecal samples in this study suggests a rather alarming situation given that this animal, popularly known as feral boar or feral pigs, is considered to be one of the biggest bioinvaders in South America [49]. Its presence can give rise to competition for ecological niches, which is detrimental to other species of the national fauna. This could probably be causing native species to seek other locations outside the park. This fact may have made it difficult to find feces from other native artiodactyl species in the areas of the park.

Nucleotide sequences compatible with *M. gouazoubira* (gray brocket) were identified in only two samples collected in Morro Redondo. The difficulty in amplifying the DNA and the low identity of the nucleotide sequences of the host obtained from the fecal samples collected in Morro Redondo may have been due to the fact that they were found in an extremely dry environment since, at the time of collection, the vegetation in this area had recently been burned in a forest fire. This condition may have degraded the DNA of interest, and the parasites present in the samples too. It should also be noted that the low identity found in the sequences of this study with the reference deposited in GenBank may be attributed to the high genetic variation that has been reported in Neotropical deer, based on the analysis of the mitochondrial genome, in a comparison of the genetic material of these animals from different locations [50]. This difficulty is confirmed by the paucity of parasitological studies found in the literature on this animal, highlighting one conducted on a private reserve in Bolivia and two in Brazil, one of them at a Rehabilitation Center, and the other in a Conservation Reserve [51,52,53].

In general, a positivity rate of 53.4% for gastrointestinal parasites was detected in the fecal samples collected in the park and its buffer zones. Higher positivity rates than this have been reported in other studies that investigated parasites in artiodactyl feces. This was the case of artiodactyl feces collected in other conservation units in Brazil, including the Private Reserve of Natural Heritage of SESC Pantanal (SESC/RPPN) in the state of Mato Grosso, and in Itatiaia National Park, located in the states of Rio de Janeiro and Minas Gerais, where positivity rates of 73.2% (41/56) and 87.1% (27/31) for gastrointestinal parasites were detected by spontaneous sedimentation and using both sedimentation and flotation techniques, respectively [20,46]. Case records also show higher parasite positivity rates in feral boar in Federal Conservation units in Mexico, where 84.4% (76/90) positivity was found using direct examination and centrifuge technique flotation and 100% (60/60) positivity in feces of several species of deer using the flotation technique, as well as in fecal matter from free-living wild boar in Iran, 61.9% (13/21) by the techniques of sedimentation and flotation, and 67% (8/12) using direct examination and centrifugal sedimentation [48,54,55,56].

It is worth highlighting that unlike the others, this was the first study that analyzed in-depth gastrointestinal parasites in fecal samples from free-living artiodactyls obtained in a non-invasive way, combining microscopic and molecular techniques to identify and characterize a species of protozoan that has the potential for zoonotic transmission and can impact public health. Although there was a high frequency of positive samples, it is important to highlight that many of the parasites detected may be cases of pseudoparasitism or even contamination of fecal samples with helminths or protozoa of the environment.

The frequency of parasites in more than half the fecal samples collected from free-living wild artiodactyls was expected since the park and its buffer zones have environments rich in biotic and abiotic factors, providing ideal conditions for the development and maintenance of parasitic structures and thus favoring the constant reinfection of fauna by these biological agents. It is important to point out that there are several large and small farms and other homesteads that produce root and tuber crops and have orchards and forage pastures in the buffer zones of the park and in areas bordering the forest of Visconde de Mauá, RJ. This wealth of homesteads ends up attracting wild artiodactyls, mainly feral boars, bringing them into closer proximity to domestic animals and humans, which may favor the exchange of parasitic agents.

In general, the sample size of fecal matter identified as belonging to *S. scrofa* proved to be sufficient within the previously planned collection period. This sufficiency was achieved with the collection of 36 fecal samples, since parasite richness, i.e., different forms of parasites, were no longer detected starting from this quantity, leading to the formation of a plateau in the parasite accumulation graph. Unlike this study, the sample size sufficiency of feral pig feces was not reached in fecal samples collected non-invasively in Serra da Capivara National Park, state of Piauí—PI and in the Itatiaia National Park, RJ and MG, because much fewer samples were collected there, i.e., 8 and 12 samples, respectively [20,45]. The number of samples collected in the park, RJ, thus led to a higher parasite richness identified there (R = 8) than the richness indices reported in other studies (R = 6). Although the sample size of *S. scrofa* feces was generally sufficient, a quantitative analysis of these samples stratified by valleys indicated that this sufficiency was reached in Pavão Valley, which was the preponderant place to obtain the parasite richness generated from the 36 samples. The non-stabilization of the parasite accumulation curve in Grama Valley seems to be directly due to the greater difficulty the research team experienced in finding artiodactyl feces in that area.

The higher indices of parasitic diversity in the feces of *S. scrofa* collected in Pavão Valley than in those collected in Grama Valley underscored the fact that the frequencies of parasites detected in the samples of these animals differed according to their roaming sites. The higher diversity indices found in Pavão Valley stem not only from the previous successful monitoring of the area but also, and mainly, from the fact that this valley is the site of the oldest establishment of a feral pig population in the area. It should be noted that Pavão Valley borders the Itatiaia National Park, possibly the place of origin of these animals, where free-living pigs have been recorded since 2012 through photographs, specifically in the area of Vargem Grande and Serra Negra [57]. However, these animals were first recorded in the park by park managers only in 2017 between Pavão and Cruzes Valleys in the same mountain range as Itatiaia National Park, a Federal Conservation unit that borders Pedra Selada State Park.

Cysts of the phylum Ciliophora stood out among the parasitic forms most frequently detected in the feces of artiodactyls in this study in both Pavão and Grama Valleys. These were also the most numerous parasite forms identified in the feces of animals identified as *S. scrofa*, predominating among the parasitic forms recovered in the feces of these animals collected in Grama Valley. The identification of cysts of this phylum in the feces of these animals was expected since pigs are considered the main reservoirs of the ciliated protozoan *B. coli* [14,16]. However, the identification of this parasite can only be confirmed using molecular techniques [33].

In this study, all the samples containing cysts of the phylum Ciliophora 39.6% (40/101) were subjected to molecular characterization. However, not all of them generated PCR-amplified DNA, nor electropherograms that could be properly interpreted 35% (14/40). The lack of 100% success in the PCR and in sequencing was to be expected since field samples obtained non-invasively may have a low DNA quality because of the weathering they undergo when they remain in the environment for a long time [58]. This difficulty may be even greater when analyzing the scat of free-living animals, particularly artiodactyls, which were the focus of this study since they ingest large amounts of plant fibers that can hinder the PCR amplification of DNA.

Thus, the 26 samples containing cysts of the phylum Ciliophora that generated nucleotide sequences that could be interpreted were characterized as *B. coli*. These sequences showed high levels of identity when compared with the reference sequences of *B. coli* isolated and maintained in xenic cultures from pig feces collected at a technified farm in Brazil, as well as sequences obtained from captive chimpanzee feces in Cameroon, from a human patient from Bolivia, from wild boar and domestic pigs in the Czech Republic and Central Africa, respectively, and also from domestic pigs, an ostrich, and a gorilla in Spain [31,32,33,58]. Thus, based on a phylogenetic analysis using the molecular marker ITS1.5.8S.ITS2, the generated sequences were found to be grouped with nucleotide sequences of *B. coli* originating from other host species in different countries, emphasizing their low host specificity and possibly a wide geographic distribution of the protozoan.

Only a few of the articles retrieved in the literature confirm the presence of *B. coli* in free-living feral pig feces identified using molecular biology techniques. Similar to this study, *B. coli* was also identified by means of molecular tools in the feces of free-ranging feral pigs in Iran [59]. Thus, it should be noted that this is the first study conducted in the Americas that identified the zoonotic protozoan *B. coli* by means of the molecular analysis of a biological sample from free-living *S. scrofa*, indicating that this bioinvader is a potential reservoir of the protozoan in a tropical region.

With regard to the genetic variants identified in the nucleotide sequences of *B. coli* in the feces of the artiodactyls in the Pedra Selada State Park, the majority consisted of type B0, followed by type A0. The literature consulted for this study revealed that type B0 has previously been reported to infect domestic pigs and an ostrich in molecular epidemiology studies in Spain and domestic pigs in the Central African Republic, China, and Korea [32,33,60,61]. On the other hand, type A0 has been identified not only in this study but also in fecal samples from captive wild boars in the Czech Republic, domestic pigs in Brazil and Spain, in non-human primates in Africa, as well as in human feces in Bolivia [31,33,58].

It should be noted that in this study, type A0 was the most frequently identified nucleotide sequence in the feces of free-ranging feral boar in Pavão Valley, specifically where there are more homesteads than in Grama Valley. This finding caused some concern, as this variant has been identified in the literature as having a zoonotic potential, since it has previously been characterized in human feces from Bolivia [58]. However, there are still only a few nucleotide sequences of *B. coli* originating from human biological material deposited in public databases containing molecular information. This information underscores the need for further studies of human biological samples, especially in countries like Brazil, where human balantidiasis has frequently been reported [12].

In addition to the abovementioned types of genetic variants, a variant classified as type B1 was also found. Despite rare reports of this variant in the literature, it has previously been reported in pig and gorilla feces in Spain [60]. A variant designated as type A was also found, although it is closer to the A0 type, it presented nucleotides also inherent to the A1 and A2 variants in helix II of the ITS1. Both type B1 and non-subtype A may have occurred due to the greater diversity of the genetic variants of *B. coli* that may be infecting boar–pig crossbreed in the region. It should be noted that pigs are omnivorous, coprophagous animals that have the habit of rooting [3,62]. These traits render them highly susceptible to infection by *B. coli* cysts with different type-variant profiles, especially in the case of free-living animals. Moreover, the protozoan in question is able to exchange genetic material through conjugation, which may have culminated in the atypical profile of type A.

Other forms of parasites were also detected in the feces of artiodactyls, but most of them could only be identified since the host animals were free-living in the wild and subject to possible infection by unknown parasite species. Among the parasites identified, both *Ascaris* spp. and *Metastrongylus* spp. were detected in the feces of *S. scrofa*. *Ascaris* spp. eggs have also been found in biological material from feral pigs in a national reserve in Spain, in national parks, a private reserve, on pig breeding farms in Brazil, as well as in a province in Iran [20,43,45,46,55]. On the other hand, *Metastrongylus* spp. were found not only in this study but have also been reported in material from feral pigs in national reserves in Spain, in provinces in Iran, private reserves in Brazil, and conservation units in Mexico [43,46,47,48]. The discovery of *Metastrongylus* spp. only in feces collected in Pavão Valley may be ascribed to the larger number of samples collected there, and even to the presence of annelids in this niche, which are intermediate hosts essential for the maintenance of this parasite’s biological cycle. The diagnosis of *Ascaris* spp. and *Metastrongylus* spp. in the feces collected in the park reinforces that they may belong to the Suidae family, even those that could not have their host identified, since these parasites are generally identified in the feces of these animal species. The diagnosis of the eggs of these nematodes demonstrates that the park’s environment is contaminated with evolutionary forms of other parasites such as nematodes, which can be transmitted for other animal species and also to humans, since *Ascaris* spp. and *Metastrongylus* spp. also have zoonotic potential.

It should be noted that rough-shelled eggs resembling those of *Ascaris* spp. were found only in fecal matter identified as *M. gouazoubira* in Morro Redondo. Although no description of ascarid species infecting cervids was found in the literature, a possible infection of this nematode in this group of animals was also reported in *Mazama temama* and *Odocoileus virginianus texanus* in a conservation unit in Mexico [56]. Because it is an animal with few parasitological studies and rarely found in the Atlantic Forest, the detection of this parasitic form deserves further investigation in order to determine if the taxon normally infects these animals or if it may have been introduced into this biome through environmental contamination via scat from other animal species, including the bioinvasive feral boar.

Other parasitic forms were also detected in artiodactyl feces collected in Pavão and Grama valleys, including typical forms of pig parasites such as thin-shelled eggs morphologically similar to those of strongyles, nematode larvae, eggs of *Trichuris* spp., and unsporulated coccidian oocysts. Parasitic forms of the aforementioned nematodes have also been detected in the feces of roaming pigs in the private reserve in Brazil’s Pantanal wetlands, in Northern Iran, and in a conservation unit in Mexico [46,48,55]. These parasitic larvae forms may belong to the group of strongyles regarded as belonging to the superfamilies Trichostrongyloidea and Strongyloidea, or even the superfamily Rhabditoidea, which includes *Strongyloides ransomi* and *Globocephalus urosubulatus*. The identification of these larvae as belonging to strongyles is reinforced by the detection of thin-shelled eggs also identified in the feces of *S. scrofa*, which were morphometrically compatible with those of this group of nematodes.

In addition, the size of the eggs of *Trichuris* spp. and of the non-sporulated oocysts of the identified coccidia was larger than those described in the literature [63] for the species that frequently infect domestic pigs. The size of the oocyst detected in the feces of *S. scrofa* in this study was morphometrically similar to the description of a typical sporulated oocyst of *Eimeria* spp., also found in the feces of this bioinvader collected in Itatiaia National Park [20]. It should be noted that this conservation unit borders the Pedra Selada State Park, indicating that these oocysts may actually belong to the genus *Eimeria*.

In addition to these parasites, a taxonomically unidentified nematode egg was also detected in a fecal sample from *S. scrofa* collected in Pavão Valley. This parasitic form even resembled an ascarid, but it was not typical of eggs of the genera that normally infect animals, such as *Ascaris* spp., *Toxocara* spp., *Toxascaris* spp., *Parascaris* spp., and *Neoascaris* spp. Although it may belong to a parasitic species that has not yet been identified, one cannot ignore the fact that this species and the others of sizes unlike those described in the literature can actually be attributed to cases of pseudoparasitism.

## 5. Conclusions

Although a population density study was not carried out, the findings of this study confirmed the presence of the bioinvading artiodactyl *S. scrofa*, popularly known as feral boar or “javaporco” roaming in Pedra Selada State Park, a state conservation unit located in the Atlantic Forest biome. Since several fecal samples from these animals were recovered in areas of the park, this bioinvader can cause physical damage by depredation of the biome, economic losses by the destruction of local farm produce, and impacts on public health through the transmission of pathogens to native fauna and residents of the region [62,64]. This biological impact was clearly evidenced, since parasites with zoonotic potential were diagnosed in the feces of this bioinvader, especially *Ascaris* spp., *Metastrongylus* spp., but mainly *B. coli* with different genetic variants, including type A0, which has previously been reported to infect humans. These parasites can give rise to severe lung and intestinal problems in humans, including dyspnea and dysentery [10,11,12,13]. It should be noted that the topographic relief of the Pedra Selada State Park, which comprises mountains and slopes, and the humid tropical climate of the state of Rio de Janeiro, are natural conditions that favor contamination and environmental dispersion, through leaching, of the resistant structures of these parasites, as attested by the eggs and cysts detected in this study. This picture underscored a problematic situation resulting indirectly from human actions that favored the presence of feral boars in conservation units in Brazil [58]. This complex situation can only have positive outcomes if it is addressed using a One Health approach since it will require efforts, monitoring, and the engagement of professionals from different areas, managers, and awareness of society, since the presence of this bioinvader may be detrimental to environmental, human, and animal health, particularly to local biodiversity. We would like to emphasize that future studies, with deeper research, should be carried out in these and other conservation units within the One Health approach in order to prevent human and wild animal infections of local fauna.

## Figures and Tables

**Table 1:** Frequency of gastrointestinal parasite structures detected in fecal samples from animals of the order Artiodactyla collected in Pedra Selada State Park, RJ, and in its buffer zones (BZs).

Parasites	Pavão Valley (n = 72)		Grama Valley (n = 24)		Morro Redondo (n = 5)		Total of Samples (n = 101)	*p* Value
	*Sus scrofa* (n = 60)	UIS artiodactyl (n = 12)	*Sus scrofa* (n = 19)	UIS Artiodactyl (n = 5)	*Mazama gouazoubira* (n = 1)	UIS Artiodactyl (n = 4)		
Protozoa	29 (48.3%)	2 (16.6%)	9 (47.3%)	1 (20%)		-	41 (40.5%)	
Phylum Ciliophora cyst	27 (45%)	2 (16.6%)	10 (52.6%)	1 (20%)	-	-	40 (39.6%)	0.00 *
Unsporulated coccidian oocysts	3 (5%)	-	-	-	-	-	3 (2.9%)	0.243
Helmints	22 (36.6%)	2 (16.6%)	3 (15.7%)	-	1 (100%)	-	28 (27.7%)	
*Ascaris* sp.	9 (15%)	-	2 (10.5%)	-	1 (100%)	-	12 (11.8%)	0.004 *
*Metastrongylus* sp.	5 (8.3%)	1 (8.3%)	-	-		-	6 (5.9%)	0.027 *
Strongyle	4 (6.6%)	-	-	-		-	4 (3.9%)	0.118
*Trichuris* sp.	4 (6.6%)	-	-	-		-	4 (3.9%)	0.118
Nematode larva	6 (10%)	1 (8.3%)	1 (5.2%)	-		-	8 (7.9%)	0.005 *
Unidentified nematode egg	1 (1.6%)	-	-	-		-	1 (1%)	1
Total parasite positivity							54 (53.4%)	

Strongyle: Eggs of the superfamilies Trichostrongyloidea and Strongyloidea. UIS (unidentified species); * *p* value ≤ 0.05. The evolutionary form of the parasite was not detected.

**Table 2:** Richness, dominance, and diversity of gastrointestinal parasites detected in *Sus scrofa* feces collected in Pedra Selada State Park and its buffer zones, Rio de Janeiro.

*Sus scrofa*	Richness	Dominance	Diversity			
			Shannon (H’)	*p* Value	Simpson (D)	*p* Value
Pavão Valley	8	2.623	1.672	0.001 *	7.377	0.041 *
Grama Valley	3	6.213	6.871		3.787	

* *p* value ≤ 0.05.

## Data Availability

The data that support the findings of this study are available from the corresponding author upon reasonable request.

## Supplementary Materials

The following supporting information can be downloaded at: https://www.mdpi.com/article/10.3390/ani13233611/s1, Table S1. Description of parasitic structures detected in samples of artiodactyl feces collected in Pedra Selada State Park and its buffer zones, Rio de Janeiro, Brazil; Figure S1. Photographs of the evolutionary forms of the parasites detected in fecal samples collected in Pedra Selada State Park, RJ, and its buffer zones. A—Protozoan cyst of the phylum Ciliophora. B—Egg of *Ascaris* spp. and protozoan cyst of the phylum Ciliophora. C—Cyst of the phylum Ciliophora. D—*Trichuris* spp. E—Egg of *Metastrongylus* spp. F—Taxonomically unidentified nematode egg. A,B,D,E,F photographs at 400× magnification (Bar 40 µm) and C—photograph at 100× magnification (Bar 25 µm). Source: The author.
