# Supplementary material for: Zoonotic Parasites in Artiodactyls with Emphasis on the Feral Boar in the Atlantic Forest, State of Rio de Janeiro, Brazil
Source: Animals (Basel). 2023 Nov 22;13(23):3611. doi: 10.3390/ani13233611 (PMC10705221; doi:10.3390/ani13233611)
Supplement: Supplementary file 1 [file animals-13-03611-s001.zip › Figure S1.pdf]

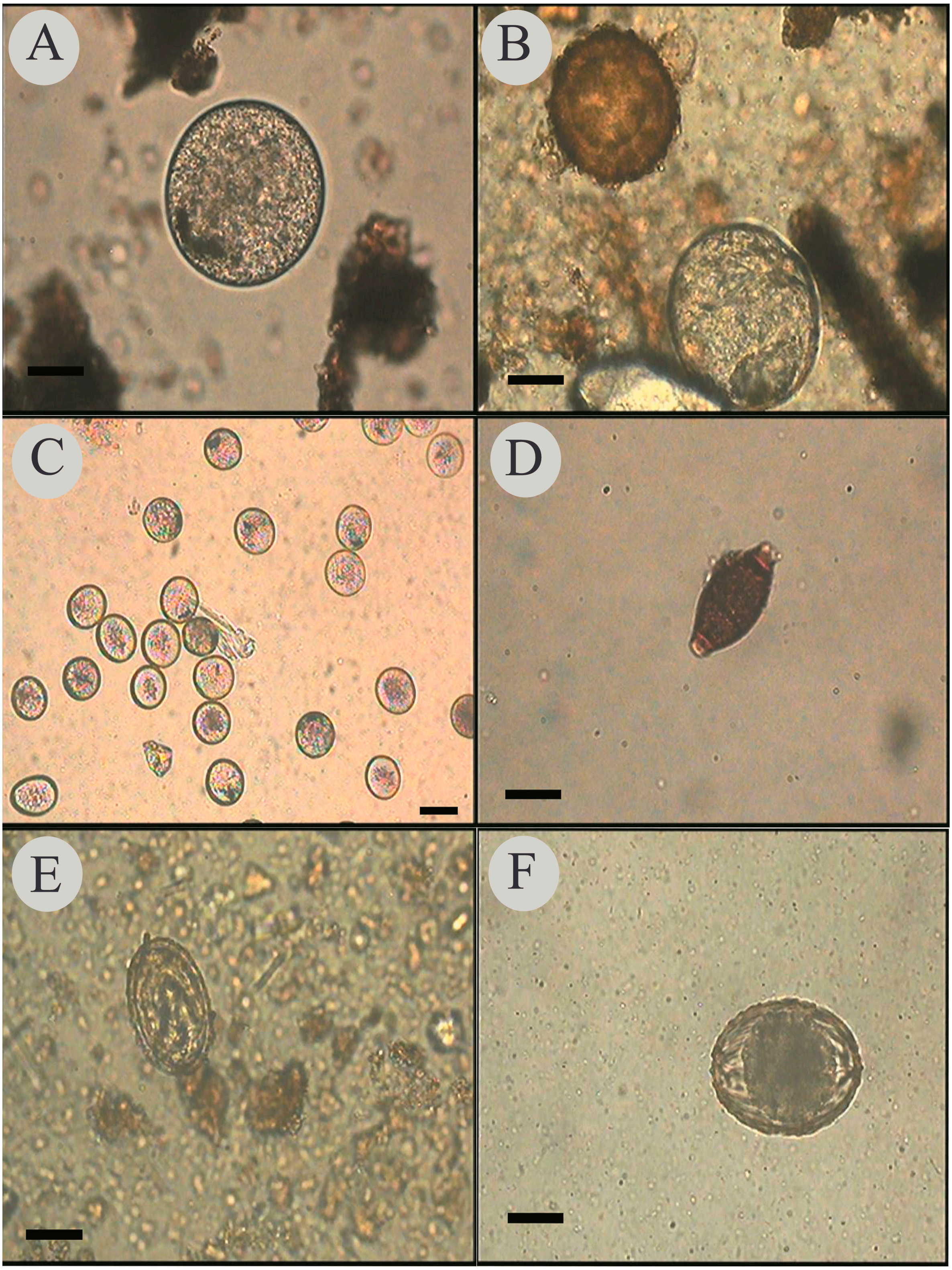

Figure S1. Photographs of the evolutionary forms of the parasites detected in fecal samples collected in Pedra Selada State Park, RJ, and its buffer zones. A—Protozoan cyst of the phylum Ciliophora. B—Egg of *Ascaris* spp. and protozoan cyst of the phylum Ciliophora. C—Cyst of the phylum Ciliophora. D—*Trichuris* spp. E—Egg of *Metastrongylus* spp. F—Taxonomically unidentified nematode egg. A,B,D,E,F photographs at 400× magnification (Bar 40 μm) and C—photograph at 100× magnification (Bar 25 μm). Source: The author.
